# Giant Postoperative Seroma after Laparoscopic Obturator Hernia Repair: A Case Report

**DOI:** 10.70352/scrj.cr.26-0388

**Published:** 2026-07-09

**Authors:** Yuki Sakamoto, Masaya Matsumoto, Ryota Omura, Katsunobu Taki, Nobutaka Sato, Shinichi Akahoshi

**Affiliations:** Department of Gastroenterological Surgery, Kumamoto Kenhoku Hospital, Tamana, Kumamoto, Japan

**Keywords:** obturator hernia, seroma, surgery, postoperative complication

## Abstract

**INTRODUCTION:**

Obturator hernia is frequently seen in thin, elderly women, and is often diagnosed following incarceration. Postoperative seroma formation is a well-recognized complication after inguinal hernia repair. In contrast, reports of seroma formation following obturator hernia repair are scarce, and its clinical features have not been well characterized.

**CASE PRESENTATION:**

A 94-year-old woman presented with nausea and vomiting and was diagnosed with an incarcerated left obturator hernia. Manual reduction was successful and was followed by elective laparoscopic repair using the totally extraperitoneal approach with mesh placement. Although the initial postoperative course was uneventful, a large preperitoneal seroma compressing the urinary bladder developed on POD 23 and required percutaneous aspiration. The seroma subsequently became infected, necessitating repeated drainage and ultimately laparoscopic mesh removal. Despite temporary stabilization, the patient experienced sudden cardiopulmonary arrest and died postoperatively.

**CONCLUSIONS:**

Postoperative seroma is a complication that can occur following surgery for an obturator hernia. Because obturator hernias are frequently seen in elderly patients with poor nutritional status, careful perioperative management is essential, bearing in mind that postoperative complications can have fatal consequences.

## INTRODUCTION

Obturator hernia is a rare type of abdominal wall hernia and is frequently associated with incarceration. Surgical repair is the standard treatment for incarcerated obturator hernia. However, because this condition predominantly occurs in elderly, thin women,^1)^ careful judgment is required when determining the indication and timing of surgical intervention.

Postoperative seroma formation is a well-recognized complication after inguinal hernia repair. In contrast, reports of seroma formation following obturator hernia repair are scarce, and its clinical features have not been well characterized.

Herein, we report a case of a giant postoperative seroma that developed after an elective laparoscopic repair performed following successful manual reduction of an incarcerated obturator hernia, along with a review of the relevant literature.

## CASE PRESENTATION

A 94-year-old woman presented to the emergency department with nausea and vomiting. She was diagnosed with an incarcerated left obturator hernia, and manual reduction was successfully performed. Preoperative assessment revealed that the patient was independent in activities of daily living, with a performance status of 1. Her medical history included old myocardial infarction, diabetes, hypertension, dyslipidemia, and osteoporosis. Her physical status was classified as American Society of Anesthesiologists class III. She was small and thin, with a height of 140 cm, body weight of 24.9 kg, and a BMI of 12.7 kg/m^2^. Laboratory findings showed a serum albumin level of 4.1 g/dL, a total protein level of 6.4 g/dL, and a lymphocyte count of 719/μL, suggesting a degree of immunonutritional impairment. Three days later, laparoscopic obturator hernia repair was carried out using the totally extraperitoneal (TEP) approach. Intraoperative findings revealed a concomitant femoral hernia. A 15 × 10-cm self-gripping mesh was placed in the preperitoneal space to cover the obturator foramen. The postoperative course was uneventful, and she was discharged home on POD 5.

**Fig. 1:**
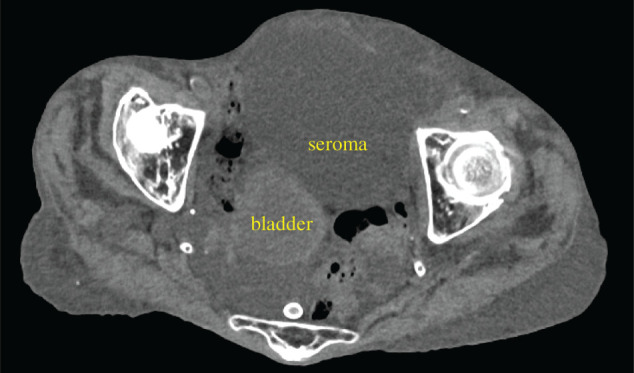
CT on POD 23 demonstrating a large postoperative seroma (approximately 12 × 12 cm) in the left preperitoneal space, compressing the urinary bladder.

On POD 23, the patient re-presented to the emergency department with left lower abdominal swelling and urinary frequency. CT revealed a large postoperative seroma, approximately 12 × 12 cm, in the left preperitoneal space compressing the urinary bladder (**Fig. 1**). A single-session percutaneous needle aspiration was performed, yielding approximately 180 mL of serous fluid.

**Fig. 2:**
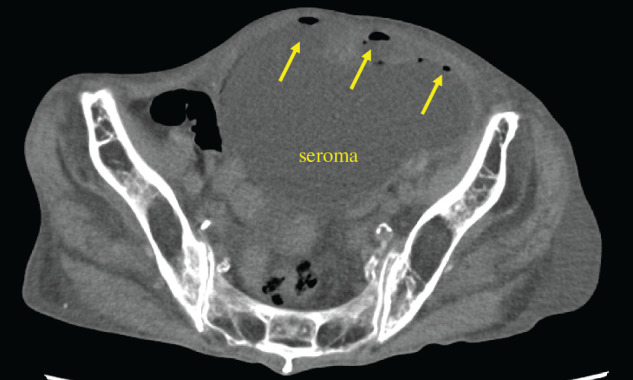
CT on POD 61 demonstrating the previously identified seroma with newly developed intralesional air (arrows), which was absent on prior imaging.

On POD 61, she again presented with fever and lower abdominal pain. CT demonstrated persistence of the seroma with newly developed intralesional air, which had not been present on the prior imaging (**Fig. 2**). On the same day, percutaneous needle aspiration yielded approximately 700 mL of yellowish, cloudy fluid, and an infected seroma was diagnosed. Culture of the drainage fluid identified extended-spectrum β-lactamase–producing *Escherichia coli*. Intravenous meropenem was administered from day 61 to day 78, followed by sulbactam/ampicillin from day 79 to day 89. Ten days after the initial drainage, recurrence of the seroma was observed, and a percutaneous drainage catheter was placed. Although inflammatory markers showed a tendency to improve, the catheter was accidentally self-removed 9 days after placement. Approximately 1 month after initiation of conservative treatment for the infected seroma, complete resolution was considered unlikely with nonsurgical management alone. As time passed, the patient’s nutritional status deteriorated; laboratory results showed total protein levels of 5.1 g/dL, serum albumin levels of 2.1 g/dL, and a lymphocyte count of 660/μL, all of which had worsened compared to the values prior to the initial surgery. Therefore, laparoscopic mesh removal was performed on POD 85 following the initial hernia repair.

**Fig. 3:**
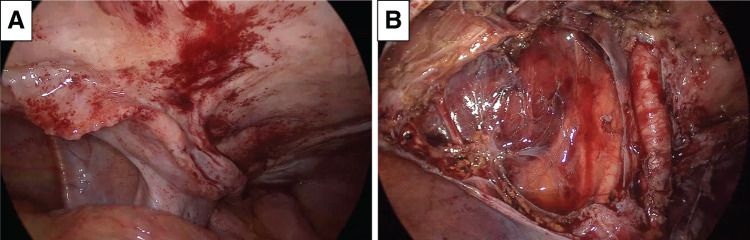
(**A**) Laparoscopic view before mesh removal, showing the mesh firmly adherent to the peritoneum and forming part of the abscess wall. (**B**) Laparoscopic view of the inguinal floor after removal of the mesh and the attached peritoneum. The peritoneal defect was left open.

Because a preperitoneal approach was technically challenging, the mesh was accessed and removed from the intraperitoneal side. Intraoperatively, the mesh was firmly adherent to the peritoneum and formed part of the abscess wall; it was therefore carefully removed together with the attached peritoneum (**Fig. 3**). Since an infection was detected, the peritoneal defect was left open to prevent the formation of a contaminated closed space. Two drains were placed in the preperitoneal space to ensure adequate drainage, and the procedure was concluded.

The postoperative course was initially stable. However, in the early morning of POD 7 after mesh removal, the patient developed hematochezia and vomiting, followed by sudden cardiopulmonary arrest. Resuscitation was attempted, but return of spontaneous circulation was not achieved, and the patient died. The cause of death was considered to be choking secondary to vomiting, likely associated with decreased intestinal peristalsis due to ischemic colitis.

## DISCUSSION

Obturator hernia is a rare type of abdominal wall hernia, accounting for approximately 0.07% to 1.00% of all hernia repairs, and it predominantly affects elderly women.^1)^ Despite this characteristic patient population, postoperative seroma formation after obturator hernia repair has not been well characterized, and specific incidence data remain lacking. By contrast, postoperative seroma has been more frequently reported after laparoscopic inguinal hernia repair, with reported incidence rates ranging from approximately 0.5% to 13.0%.^2,3)^

The apparent rarity of seroma formation after obturator hernia repair may be partly explained by the generally small size of the hernia sac compared with inguinal hernias. A smaller hernia sac may leave less residual space after reduction, thereby reducing the likelihood of postoperative fluid accumulation. However, this explanation may not fully apply to laparoscopic obturator hernia repair, in which the extent of preperitoneal dissection is comparable to, and sometimes greater than, that required for laparoscopic inguinal hernia repair.

Given the lack of specific data for obturator hernia, insights from laparoscopic inguinal hernia repair may help clarify potential mechanisms of seroma formation. Previous studies have demonstrated that extensive preperitoneal dissection and the creation of dead space facilitate the accumulation of inflammatory exudate following surgical trauma and mesh placement.^4,5)^ In addition, preoperative incarceration requiring manual reduction has been suggested to increase the risk of seroma formation by inducing persistent local inflammation and tissue edema.^6)^ Retrospective analyses have further identified larger hernial orifice size, use of the TEP technique, and low preoperative serum albumin as independent risk factors, indicating that both surgical and host-related factors contribute to seroma development.^5)^ Moreover, systematic reviews suggest that the method of hernia sac management may influence seroma incidence by leaving residual anatomical spaces prone to fluid collection.^7)^

Taken together, these findings suggest that postoperative seroma formation is not determined solely by the size of the hernia sac. Even in obturator hernia repair, seroma formation may occur when extensive preperitoneal dissection is combined with unfavorable host factors, such as advanced age and poor nutritional status, which may impair inflammatory resolution and tissue adhesion. In the present case, the need for preoperative manual reduction of the incarcerated hernia and the patient’s poor nutritional status were considered to have contributed to postoperative seroma formation. Advanced age and poor nutritional status have consistently been associated with impaired wound healing, delayed inflammatory resolution, and increased susceptibility to postoperative infection.^8)^ In frail elderly patients, even a localized fluid collection such as a seroma may therefore progress to clinically significant infection.

In this case, the patient ultimately died following a complicated postoperative course. Considering the possibility of a concomitant inguinal hernia, we opted for laparoscopic TEP repair with mesh coverage of the myopectineal orifice. Indeed, a femoral hernia was confirmed intraoperatively, confirming the need for comprehensive reinforcement using a large mesh. On the other hand, the extensive dissection may have contributed to the development of a seroma; in cases with poor preoperative nutritional status such as this one, less extensive dissection, smaller mesh coverage, or limited obturator repair might have been preferable. Furthermore, since this was the patient’s first episode of incarcerated obturator hernia, the option of observing the condition without performing curative surgery was also available.^9)^ However, surgery must be considered in cases of recurrent incarceration or if the patient requests it. In this case, prior to the initial surgery, the patient and primary family members were provided with a detailed explanation of the risks of complications, including fatal outcomes, and informed consent was obtained.

Because an obturator hernia often involves a high risk of perioperative complications, as seen in this case, careful preoperative evaluation and selection of an individually optimized surgical strategy are essential for determining the treatment plan.

## CONCLUSIONS

This case highlights that even obturator hernias with small hernia sacs may develop a postoperative infected seroma after laparoscopic repair in frail elderly patients with poor nutritional status. Careful perioperative management is essential, bearing in mind that postoperative complications can have fatal consequences.

## DECLARATIONS

### Availability of data and materials

All relevant data are included in this article.
